# Small Cyclic Peptide for Pyrophosphate Dependent Ligation in Prebiotic Environments

**DOI:** 10.3390/life10070103

**Published:** 2020-07-02

**Authors:** Radosław W. Piast, Maciej Garstka, Aleksandra Misicka, Rafał M. Wieczorek

**Affiliations:** 1 Faculty of Chemistry, University of Warsaw, Pasteura 1, 02-093 Warsaw, Poland; rpiast@chem.uw.edu.pl (R.W.P.); misicka@chem.uw.edu.pl (A.M.); 2 Department of Metabolic Regulation, Faculty of Biology, University of Warsaw, Miecznikowa 1, 02-096 Warsaw, Poland; garstka@biol.uw.edu.pl

**Keywords:** origin of life, peptides, pyrophosphate, prebiotic ecology, metallopeptides

## Abstract

All life on Earth uses one universal biochemistry stemming from one universal common ancestor of all known living organisms. One of the most striking features of this universal biochemistry is its utter dependence on phosphate group transfer between biochemical molecules. Both nucleic acid and peptide biological synthesis relies heavily on phosphate group transfer. Such dependents strongly indicate very early incorporation of phosphate chemistry in the origin of life. Perhaps as early as prebiotic soup stage. We report here on a short cyclic peptide, c(RPDDHR), designed rationally for pyrophosphate interaction, which is able to create a new amide bond dependent on the presence of pyrophosphate. We believe this result to be a first step in the exploration of Phosphate Transfer Catalysts that must have been present and active in prebiotic soup and must have laid down foundations for the universal bioenergetics.

## 1. Introduction

Whether one adheres more to the metabolic understanding of life [1] or more to the genetic one [2] we are still left in front of the question of the origin-of-life and how the present set of biochemical reactions came to be. A common feature of the biochemistry of all known organisms is their dependence on phosphorylated molecules as the main intermediates in bioenergetic processes [3]. As such the question of phosphorylation, availability of phosphorus, and formation of organophosphorus compounds and more specifically organophosphates has been approached multiple times by researchers interested in prebiotic chemistry and the origin-of-life process [4,5,6,7,8,9,10,11,12]. We have previously postulated [4] the existence of a hypothetical molecule named Phosphate Transfer Catalyst, which would be of crucial importance in the prebiotic emergence of biochemical pathways. Such compounds should be able to catalyze phosphorylation of diverse organic nucleophiles available in the prebiotic soup. We have argued that a metallopeptide is an ideal candidate for such a function. 

Amino acids are easily formed in diverse models of prebiotic conditions [13,14,15], they were also detected in extant extraterrestrial samples [16,17] and might have been abundantly transported to early Earth via comets [18]. Short peptides themselves have been shown to form in prebiotically plausible environments [19,20,21]. There are also numerous reports demonstrating some sort of catalytic activity of various short peptides [22,23,24,25]. Shorter, less specific and efficient peptides were probably the ancestors of modern much more specialized and efficient enzymes [26]. Modern enzymes involved in phosphate residue transfer, phosphate transferases, generally require divalent metal cations to coordinate the transition state [27]. The natural state of polyphosphates in aqueous solutions is in complexes with such ions as Mg^2+^ [28]. 

All those considerations strongly point to a short metallopeptide, likely in a complex with Mg^2+^, as the first molecule in the history of the origin-of-life process which started the phosphorylation phenomena so abundant in life. 

We decided to rationally design a number of short peptides which would exhibit low energy coordination to Mg^2+^ and to pyrophosphate. We hoped that such an approach will allow to create a Phosphate Transfer Catalysts [4] exhibiting properties allowing for further complexification of the prebiotic soup into a complex interacting mixture described as ‘prebiotic ecology’ [29]. 

Chosen peptides with the most promising theoretical features were subsequently synthetized and tested in assays of pyrophosphate hydrolysis and amide bond formation. One of those peptides, a head-to-tail cyclic hexapeptide, c(RPDDHR), named here ‘c3’, is shown to significantly increase hydrolysis of pyrophosphate as well as to create a new amide bond dependent on the presence of pyrophosphate.

## 2. Materials and Methods 

We used YASARA version 19.9.17 software for in silico design of the peptides and their subsequent energy optimization. The peptides were docked with Mg^2+^ and pyrophosphate and their energy minima were searched by molecular dynamics using Amber ff15ipq force field [30] and steepest descent energy minimization. We used periodic boundary conditions, default simulation cell size, and water molecules density 0.997 g/mL. Potential energy of an object and formation energy in water was calculated. Initially we rationally designed ca. 100 peptides among which there were present linear constructs, disulfide-bridged peptides, and head-to-tail cyclic peptides. As a crucial factor in deciding which peptides to synthesize, we chose minimal energy value, subjective ability to perform the reaction, and the ease with which the peptide can recreate the initial “working” active site after substrates were torn apart from it. 

**Figure 1:**
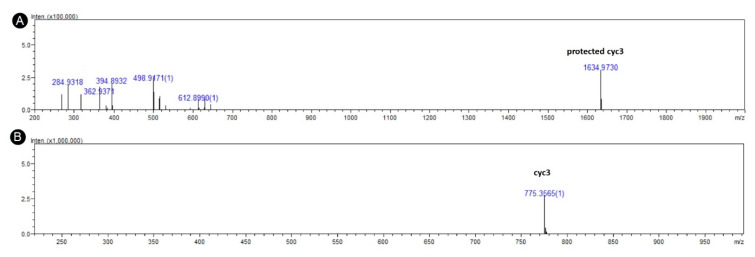
Negative ESI-TOF mass spectra of (**A**) fully protected cyc3 peptide: c(Arg(Pbf)ProAsp(OtBu)Asp(OtBu)His(Trt)Arg(Pbf)), and (**B**) the same peptide after deprotection protocol.

The most promising peptides were chosen for chemical synthesis. All the peptides were synthesized on 2-chlorotrityl resin using standard Fmoc strategy [31]. Fully protected peptides were obtained by using cleavage solution with 1% TFA in DCM and purified on c12 Jupiter Proteo column. Mass was confirmed via mass spectrometry. Cyclizations were performed by instillation of a peptide into a DMF solution with 3eq HOBT 3eq TBTU and 2% DIPEA. The reaction was performed up to 48 h and its progress was monitored on HPLC (Jupiter Proteo c12) and mass spectrometry. Peak with a mass corresponding to a fully protected cyclic peptide was isolated. Full deprotection was achieved by incubation in solution of TFA/Phenol/Water 95/2.5/2.5 *v/v* for 2 h (Figure 1). Raw deprotected peptides were purified using preparative liquid chromatography. Peak with a mass corresponding to cyclic peptide was isolated and lyophilized (Table 1). Purity was confirmed be HPLC-MS. Subsequently, counter ions were changed from TFA to chloride by dilution in 1% HCl and lyophilization. SerHis peptide was obtained from Bachem.

To determine the catalytic activity of peptides we have prepared a reaction mixture with pyrophosphate, Mg^2+^, and two amino acid derivatives (N-Me-Phe-OH-179.2 g/mol and H-Leu-NH_2_- 130.2 g/mol). The final concentration of the reagents was as follows: 1.5 mM peptide, 40 mM MES buffer pH 5.5 or 40 mM Tris buffer pH 8, 3 mM pyrophosphate, 5 mM for Mg^2+^, and 6 mM for each amino acid derivative. The reaction volume was 100 µL. Samples were incubated for 10 days at 55 °C.

The amount of phosphates (both Pi and PPi) in the soluble fraction was monitored using modified Chen’s phosphorus microdetermination method [32]. For monitoring of phosphates levels during the reaction, 1 µL from the top of the centrifuged reaction mixture was placed directly on the 96-well plate and filled up to 50 µL with miliQ water. Subsequently, 150 µL of equal volumes of 6 N sulfuric acid, 0.8 M ammonium molybdate, and 10% ascorbic acid was added. After 1 h of incubation at 60 °C the absorbance in 820 nm was measured against blind probe with water instead of sample. For determination of phosphates levels in fractions after ion exchange chromatography 50 µL of fraction was used. All the spectrophotometric assays were performed with NanoStar BMG spectrophotometer on a 96-well plate with at least two blind samples. The final result was a median from 12 measuring points per sample. Correlation of absorbance to phosphates concentration was determined through calibration with known amounts of phosphoric acid. 

To determine the amount of pyrophosphate left over in the reaction mixture we chose Dowex 1 × 8 resin. Firstly, the resin was washed with 4 M HCl and subsequently with water until the pH of the eluent reached over 4. Equal amounts of resin were divided into small 2 mL columns. The swollen resin was 15 mm in height. The reaction mixture left over after the samples were freeze-dried, was dissolved in 100 µL 10% trichloroacetic acid (TCA) and applied on the Dowex column. After 15 min of incubation the column was washed with 1 mL of water. Phosphoric acid was collected while washing the resin with 0.05% HCl while pyrophosphoric acid with 0.5% HCl. Six fractions, 1 mL each were collected. One after applying a sample and washing the resin with water, three after washing the resin with 0.05% HCl, and two after washing it with 0.5% HCl. The amount of phosphoric/pyrophosphoric acids present was determined by microdetermination of phosphorus using 50 µL of sample. 

Mass spectra were acquired on the Shimadzu LCMS-IT-TOF mass spectrometer with electrospray ionization (ESI) equipped with Jupiter Proteo column I.D. 2.0 × 250 mm, phase A: miliQ water/0.1% FA, phase B: gradient grade acetonitrile/0.1% FA, detection at 210 nm, quadrupole fixed on scan mode. Elution was with linear increase from 0% to 70% of phase B in 35 min, flow = 0.2 mL/min. 

MALDI analysis was performed on Axima Performance MALDI-TOF-TOF mass spectrometer, controlled by Biotech Launchpad 2.9.2 software (Shimadzu, Duisburg, Germany). The UV laser was operated at a pulse rate of 50 Hz. The analyzer was operated in linear mode. A total of 500 shots were accumulated for each mass spectrum acquisition. Calibrants (TofMix and PepMix2) were purchased from LaserBio Labs (Sophia Antipolis Cedex, Valbonne, France). The matrix alpha-cyano-4-hydroxy-cinnamic acid (CHCA) was used. The matrix was dissolved in water/acetonitrile (1:1, *v/v*) without any additives at 10 mg/mL. A total of 5 μL of raw sample from catalytic activity experiments was added to 5 μL of matrix solution. Such solution was then deposited onto a stainless-steel MALDI plate in a volume of 1 μL. The solvents were removed by evaporation in air at room temperature.

## 3. Results

### 3.1. In Silico Design and Synthesis

Out of ca. 100 rationally designed peptides we chose four, which showed the smallest energy of forming a complex with pyrophosphate and two Mg^2+^ ions (Table 2). The energies reported here are potential energies calculated with the force field equation. Such absolute potential energies are usually orders of magnitude larger than the relative free energies measured experimentally in the lab. They are, therefore, best described as pseudo free energies. They allow, however, for a relative comparison between different conformations. All the best peptides were cyclic ones. We believe this to be caused by the higher structural stability of cyclic hexapeptides as they are known to obtain a well-studied secondary structure of beta-sheet [33]. As such is the artifact of the screening method but it does not suggest an inherent inability of the linear peptides from performing similar functions. The search for minimal energy in the YASARA software favored molecules with lesser amounts of degrees of freedom. The four peptides were chosen for synthesis, but their tight cycle meant that their synthesis was not very efficient (<5%) and cyc2 could not be obtained at all, probably because of the presence of the PDP motive. Two prolines in close vicinity could put too much tension preventing formation of head-to-tail macrocycle. 

Three peptides, cyc3, cyc4, and cyc5, were synthesized and tested in pyrophosphate hydrolysis assay and peptide bond production assay. 

Initial pyrophosphate hydrolysis results showed that peptide cyc3 had significantly better performance than cyc4 and cyc5. For these reasons we designed and synthesized several modifications of cyc3:

- a linear version of cyc3: lin3, H-RPDDHR-OH,
- a partial alanine scan of cyc3: cyc6, c(RPDDAR); cyc7, c(RPADHR); cyc8, c(RPDAHR),
- duplication of the putative active region of cyc3: cyc9, c(RHDRHD).

### 3.2. Pyrophosphate Hydrolysis Assay

**Figure 2:**
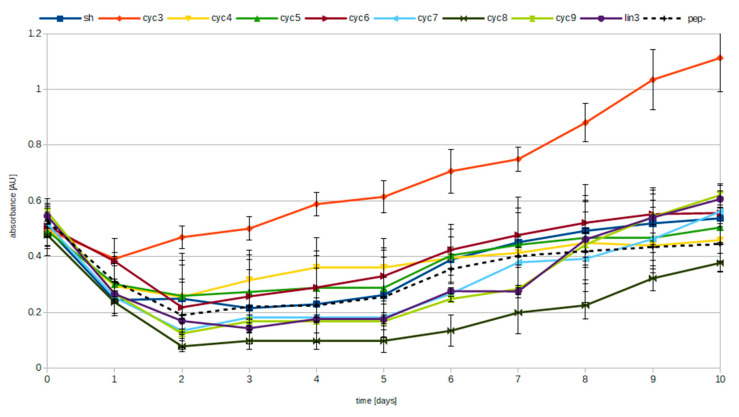
Measurements of the presence of phosphates in reactions with different peptides. Each sample was incubated for 10 days at pH 5.5 at 55 °C. Initial conditions: 1.5 mM peptide (unless negative control), 40 mM MES buffer, 3 mM pyrophosphate, 5 mM Mg^2+^, and 150 mM of NaCl. Each set of incubations was performed in triplicate. Error bars show standard deviation.

Peptides cyc3-9, lin3, and SH (the last one chosen for its broad hydrolytic activities [21]) were incubated for 10 days in the presence of Mg^2+^ and pyrophosphate. The presence of phosphate ions (PO_4_^3−^) released through the hydrolysis of pyrophosphate was monitored through phosphorus microdetermination method [32]. Figure 2 shows the relative measured amounts of phosphate ions in experiments with various peptides. Phosphorus microdetermination method responds both to phosphate ions and to pyrophosphate ions but with unequal sensitivity. For this reason, we decided to separate those ions from each other using ion chromatography and measure their relative concentration. The amount of separated phosphate and pyrophosphate ions was then determined through the microdetermination method. The results of those measurements are presented in Table 3. They demonstrate that the samples with the lowest pyrophosphate presence after the reaction (presumably due to pyrophosphate hydrolysis) do indeed contain the lesser amount of pyrophosphate inversely proportional to data presented in Figure 2.

### 3.3. Acylation of Peptide cyc3

**Figure 3:**
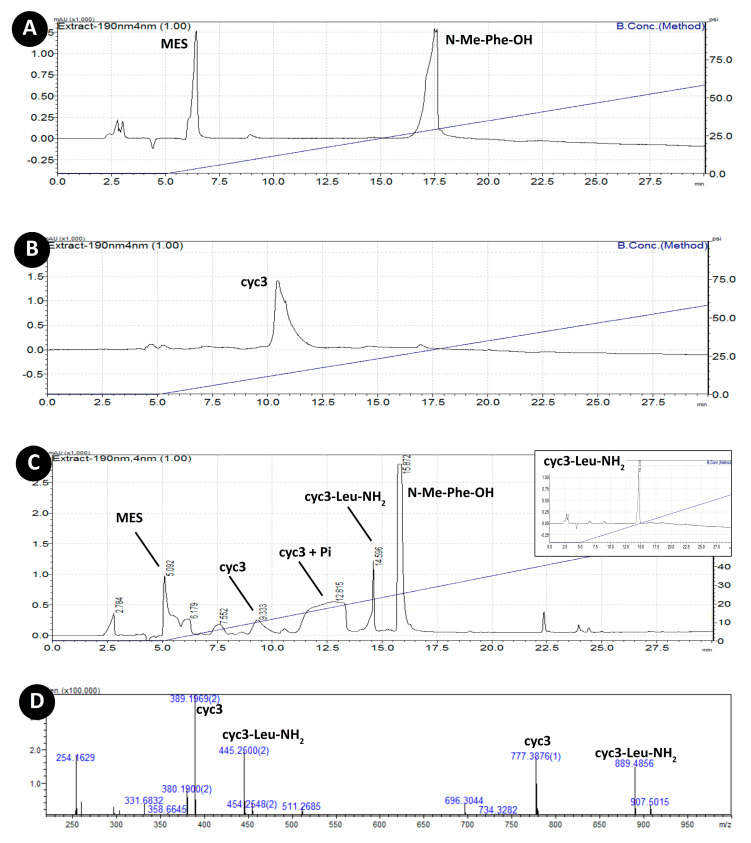

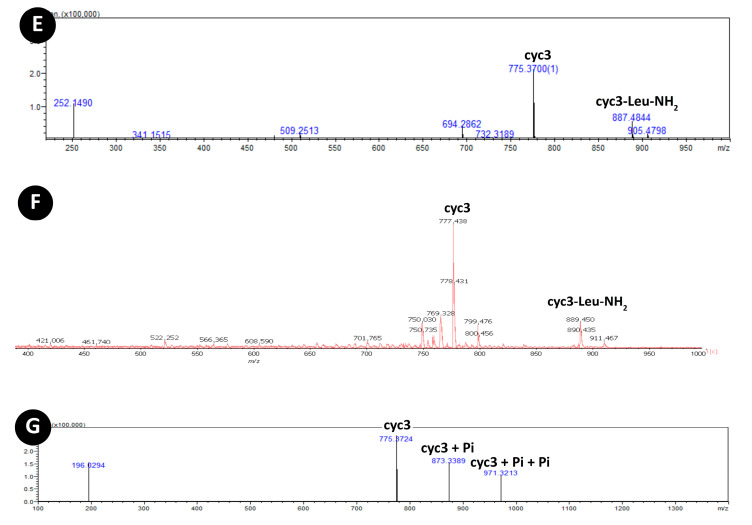
(**A**) HPLC chromatogram of reaction mixture containing Mg^2+^, PPi, N-Me-Phe-OH, and H-Leu-NH_2_. (**B**) HPLC chromatogram of cyc3 compound. (**C**) HPLC chromatogram of the same mixture as in (A) after addition of cyc3 and 10 days of incubation; insert—retention time of synthetic cyc3-Leu-NH_2_. (**D**) ESI-TOF positive ion mass spectrum of a peak with cyc3-Leu-NH_2_ mass (retention time: 14.5 min). (**E**) ESI-TOF negative ion mass spectrum of the same peak. (**F**) MALDI-TOF spectrum of the reaction mixture from (C). (**G**) ESI-TOF negative ion mass spectrum of the peak with retention time 11–13.5 min.

In addition to pyrophosphate and Mg^2+^ all peptides were incubated also in the presence of two amino acid derivatives (N-Me-Phe-OH and H-Leu-NH_2_). Only experiments with cyc3 peptide showed any interaction with the amino acids substrates. HPLC-MS analysis of cyc3 reaction mixture with Mg^2+^, pyrophosphate, N-Me-Phe-OH, and H-Leu-NH_2_ revealed the presence of a new peak with masses corresponding to cyc3-Leu-NH_2_ (Figure 3D,E) presence of such mass was additionally confirmed by MALDI-TOF (Figure 3F). To confirm its identity, we separately synthetized pure compound cyc3-Leu-NH_2_ (where Leu is connected to the cyc3 cycle through the peptide bond with the side chain of Asp4 residue). The synthetized cyc3-Leu-NH_2_ had the same mass as the compound from the reaction (data not shown) and had the same retention time as the compound from the reaction (Figure 3C insert). 

The formation of cyc3-Leu-NH_2_ compound seems to be dependent on the presence of pyrophosphate and magnesium ions as experiments with cyc3, N-Me-Phe-OH, and H-Leu-NH_2_ but without PPi or Mg^2+^ yielded no formation of cyc3-Leu-NH_2_. Presence of EDTA (in ratio 1:2 Mg^2+^: EDTA) results in suppression of the reaction too (data not shown). Additionally, cyclization of a peptide seems to be equally important as the sample with linear counterpart of cyc3 – lin3 did not yield the formation of the corresponding derivative. Additionally, we performed these experiments in three different pH ranges (4, 5.5, 8) to learn what impact the environment has on the reaction. Product cyc3-Leu-NH_2_ was present only in pH 5.5.

## 4. Discussion

We embarked on this study as an approach to a search for a Phosphate Transfer Catalyst [4], which in our opinion is crucial in prebiotic emergence of bioenergetics and of information-storing polymers. We chose a top-bottom approach reducing the active sites to merely few amino acids put on a relatively stable scaffold of cyclic heksapeptide. Of the nine peptides we used, and which were chosen for their reasonable potential for peptide bond formation linked to pyrophosphate hydrolysis, only one, cyc3, showed compelling catalytic activity.

The alanine scan showed that all of three tested residues from a putative active site (DDH) are needed for the reaction to take place. We believe that the most possible explanation of this result is the fragile active site structure. As shown by computer simulation the Asp3 coordinates Arg1 residue, Asp4 coordinates one of the magnesium ions and might also be involved in polarization interaction with the imidazole ring of His5. When Asp3 is absent, the ion bond might be formed between Arg1 and Asp4 preventing it from coordinating one of the Mg^2+^ and forming a functional substrate site for pyrophosphate.

In experiments involving pyrophosphate monitoring the initial amount of measured phosphates available in the solution has lowered after the first days because of the precipitation of Mg^2+^/PPi (Figure 2). Only with passing time levels of phosphate ions start to rise. Significantly lower levels of precipitation of magnesium pyrophosphate for cyc3 samples might be caused by strong coordination of pyrophosphate. Formation of peptide–magnesium–pyrophosphate complex might prevent Mg^2+^/PPi from precipitation. In the end low energy of such a complex is probably a reason why cyc3 has low catalytic properties as products of its reaction might also act as competitive inhibitors of that reaction.

After the initial drop in absorbance all the samples start to increase their levels of phosphates with slightly higher slope for peptide cyc3. As far as constant rise might have been a result of samples drying, the difference in slope for cyc3 could be a consequence of a catalytic activity.

We assumed that the catalysis of pyrophosphate hydrolysis most probably comes through an intermediate state in which the catalytic peptide will form a covalent bond with the phosphate group, similar to the way modern phosphate transferases, ATPases, and some synthetases work [27]. We designed the activity assay in the way that the presence of a certain product will also provide a clue to the possible mechanism by providing two amino acid substrates, one blocked at the N-end and another blocked at the C-end: N-Me-Phe-OH and H-Leu-NH_2_. Three possible outcomes of the reaction would be possible. The condensation of both amino acid derivatives N-Me-Phe-Leu-NH_2_, the addition of amino acid derivative to a peptide through carboxylic residue in the case of N-Me-Phe-OH and through amine end in the case of H-Leu-NH_2_.

In alkaline pH, no desired products were found for Me-Phe and Leu-NH_2_ as substrates. After one day the magnesium pyrophosphate precipitate was visible and no pyrophosphate hydrolysis was observed during microdetermination of phosphates. In pH 4 the magnesium pyrophosphate precipitate was not forming which resulted in phosphorus levels in the sample staying constantly high. No presence of products of any kind was observed. 

In pH 5.5 mass spectrometry showed positive results only in the case of peptide cyc3. HPLC chromatogram showed a broad peak with a mass corresponding to peptide c3 coordinating one and two phosphate residues (Figure 3G). Additionally, a peak with a mass corresponding to a cyc3-Leu-NH_2_ (888 g/mol, retention time 14.5 min) was found (Figure 3C–F). This indicates that a plausible reaction mechanism is through phosphorylation of aspartate residues. The identity of this compound was confirmed by separate synthesis of cyc3-Leu-NH_2_ (Figure 3C insert). Its structure, a cyclic head-to-tail hexapeptide, with the seventh amino acid connected through the side chain group might not be what we would tend to look for or what we would want to find in prebiotic soup but it shows us that the diversity of compounds to be considered when discussing the ‘prebiotic ecology’ [29] is great indeed. Additionally, we cannot say at present whether this diversity is a positive or negative factor in the process of the origin-of-life.

Lowering the pH from 5.5 to 4 results in lack of the cyc3-Leu-NH_2_ product. It is therefore also possible that phosphate residues are being transferred from imidazole moiety of histidine first. p*K*a of histidine residues is approximately 5.7, therefore, lowering pH would increase the pool of fully protonated imidazole ring while mildly acidic conditions would still allow existence of nucleophilic properties on one of the nitrogen atoms in this moiety. Thus, we propose that the reaction mechanism is dependent on histidine and aspartic acid interaction. One can polarize another and nucleophilically attack the phosphorus atom from pyrophosphate. Given the low potential energy of phospho-histidine derivative of cyc3, histidine residue most probably plays a part as a polarizing agent and nucleophilic attack is being performed by aspartate residue. Subsequently, if the phosphate residue is transferred on the carboxyl moiety of the aspartic acid, a substitution with one of the following nucleophile from the environment will occur: (1) hydroxyl ion (rather rare in acidic condition), may cause hydrolysis of that bond giving orthophosphate and restored cyc3 molecule or (2) leucine amide (giving a product we observe—cyc3-Leu-NH_2_). A similar reaction mechanism is commonly found in enzymes known as synthetases where carboxyl phosphates are frequently found as intermediates [34]. 

The presence of 696 *m/z* and few smaller masses in MS-TOF was puzzling to us as they might have been a result of unknown reactions. Since however, we did not observe similar masses on MALDI-TOF we consider them to be a plausible ESI generated artifacts. Additionally, mass 907 *m/z* that would suggest existence of a linear peptide ligated with Leu-NH_2_ was not found on MALDI-TOF.

**Figure 4:**
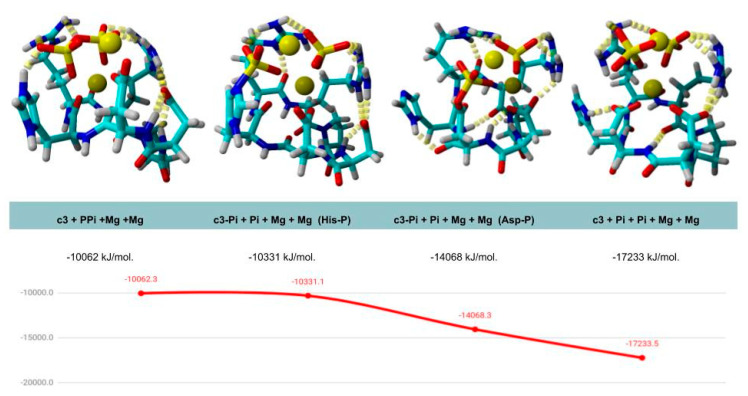
Calculated plausible intermediate structures of cyc3 peptide, two Mg^2+^ ions, and PPi that could be formed during catalysis. Energies and structures obtained in Yasara version 19.9.17 via molecular dynamics with Amber ff15ipq force field, water molecules density 0.997 g/mL, default simulation cell size with periodic boundary conditions. Energy graph suggests the most probable route of catalysis that excludes phosphohistidine intermediate from the plausible structures and provides a clue for the low activity of peptide cyc3 as product—orthophosphate in complex with cyc3 has the lowest energy and may act as a reaction’s inhibitor.

A picture of a possible mechanism emerges from our simulations of the cyc3 structure in a complex with PPi and two Mg^2+^ ions (Figure 4). 

We observe that the two arginine residues of cyc3 may anchor the pyrophosphate/phosphate (observed also in MS spectra (Figure 3G) and HPLC—retention time 11–13.5 min (Figure 3C)—through the hydrogen bonds forming a potential substrate site for this compound. 

The reaction, as shown in Figure 4, could have taken two plausible mechanisms: through phosphorylation of imidazole ring and aspartic acid residue. We modeled both of them in silico to find out which path is the most possible. Complex with a phosphoaspartate anhydrate has a significantly lower energy than a complex with a phosphoimidazole residue on the His amino acid. Last is the cyc3 peptide with a complex with magnesium ions and two orthophosphates as products of the reaction. cyc3 with a complex with magnesium and orthophosphates has the lowest potential energy which may explain why cyc3 possess catalytic properties but also why the turnover is so low. Ideally, complexes representing the transition state should have the lowest energy while in this case the product of the reaction acts as a strong inhibitor of the same reaction.

Experiments performed in pH 4 and 8 did not result in hydrolysis of pyrophosphate nor production of peptide bond, which suggests that the histidine residue and its protonation level is important for the overall action of cyc3 as imidazole ring p*K*a falls on pH 5.7 in which pH we observe products formation. It might be through the polarization of one of the aspartic acid residues by imidazole ring and subsequent attack of the carboxyl onto central phosphorus with phosphate anhydride in Asp residue production that starts the reaction.

Serine-Histidine dipeptide seems not to be active either in acidic or alkaline environments. Although multiple experiments prove the catalytic properties of this molecule (even with similar substrates) [20,23], lack of N-Me-Phe-Leu-NH_2_ product suggests SH dipeptide due to its nucleophilic properties only speeds up the ester–amide exchange reaction, since the ester of Phe was used in the previous findings.

## 5. Conclusions

In the current endeavor we aimed at finding crucial enzymatic activity of phosphate transferases in short sequence peptides. The scope of our work is origin-of-life, but we did not limit ourselves to amino acids which could be considered prebiotic. Instead we opted for possible incorporation of all natural proteinogenic amino acids to increase the low chances of finding desired activity in the chemical synthesis approach. The end result of the design project was head-to-tail cyclic hexapepides. 

Despite challenging chemical synthesis of cyclic peptides, caused by steric clashes of heavily blocked amino acids residues, such molecules might be very interesting for the origin-of-life researchers because of their secondary structure stabilization. Moreover, cyclic peptides might have been produced in prebiotic condition due to proximity of various moieties able to undergo ligation reaction while activated via numerous plausible prebiotic events. Current chemical synthesis of those compounds requires a cyclization step to be performed with all the side chains blocked by bulky protecting groups. As a result, the cyclization efficiency of such synthesis is very limited. This should not be a problem in a prebiotic soup scenario as no side chains protecting groups would impede the process of cyclization.

We designed and synthesized several short cyclic peptides with potential for pyrophosphate hydrolysis and pyrophosphate dependent peptide bond formation. Among those we found one sequence, c(RPDDHR), which exhibited catalysis of pyrophosphate dependent formation of the peptide bond. We believe that on prebiotic Earth molecules similar to c(RPDDHR), able to produce peptide bonds using energy from pyrophosphate hydrolysis-Phosphate Transfer Catalysts, were indispensable in kick-starting biochemical pathways of chemical synthesis based on the phosphorylation-substitution strategy we know from all extant living organisms. This novel mechanism bringing together ligation and chemical energy stored inside pyrophosphate bonds might have paved a way toward prebiotic anabolism and formation of even more complex molecules like nucleic acids, for instance, in which synthesis of monomers and polymers predominantly belongs to phosphate chemistry.

Such proto-enzymes could have been a milestone for origin of life on Earth providing missing catalytic properties to the prebiotic soup, giving rise to RNA world and codependency between nucleic acids and proteins known in all extant living organisms.

## Figures and Tables

**Table 1:** Peptides used in the experiments, their sequences, and masses as calculated, found on MS-TOF and MALDI-TOF.

Peptide	Sequence	Mass (g/mol)		
		Calc (g/mol)	TOF (g/mol)	MALDI (g/mol)
SH	H-SH-OH	242.23	242.1	242.26
cyc2	c(DPDPDR)	695.69	--------	--------
cyc3	c(RPDDHR)	776.82	776.38	776.49
cyc4	c(RPDDPR)	736.76	736.37	736.71
cyc5	c(DPDDPR)	695.69	695.29	695.71
cyc6	c(RPDDAR)	710.75	710.39	710.69
cyc7	c(RPADHR)	732.81	732.38	732.75
cyc8	c(RPDAHR)	732.81	732.38	732.74
cyc9	c(RHDRHD)	816.84	816.37	816.77
lin3	H-RPDDHR-OH	794.82	794.39	794.54

**Table 2:** The four peptides with the lowest energy complex formation in relation to their uncomplexed and unfolded states.

Peptide Name	Sequence	Energy of Complex Formation with PPi and 2 × Mg^2+^
cyc2	c(DPDPDR)	−9331 kJ/mol
cyc3	c(RPDDHR)	−10,062 kJ/mol
cyc4	c(RPDDPR)	−10,210 kJ/mol
cyc5	c(DPDDPR)	−9529 kJ/mol

**Table 3:** Relative absorbance of phosphates/pyrophosphates in ion chromatography fractions from a precipitate left over after the reaction. Optical density was obtained by microdetermination of phosphorus of 50 mL of eluate from each fraction. Phosphoric acid is washed out at 0.05 mM HCl (fractions 2–4) while pyrophosphoric acid is eluted at higher acid concentration—0.5 mM HCl (fractions 5–6) as shown in the last two columns with calcium phosphate and calcium pyrophoaphate salts. Peptide cyc3 sample has 50% lower amounts of pyrophosphate left over after reaction in comparison to control without peptide (-pep). Low amounts of phosphoric acid in fractions 2–4 suggest that the phosphate salts were soluble and were taken away during phosphate level monitoring done in previous steps of phosphorus microdetermination. Probe with no peptide and no magnesium ions is low on any phosphates because without divalent cations all phosphates were soluble. Colors and shades indicate the level of absorbance, red—low, green—high.

Elution Step	SH	cyc3	cyc4	cyc5	cyc6	cyc7	cyc8	cyc9	lin3	-pep	-pep -Mg	cyc3 -Mg	cyc3 EDTA	Pi	PPi
**1 H_2_O**	0.0904	0.0887	0.0538	0.0796	0.1001	0.0689	0.0562	0.1124	0.1015	0.1031	0.0987	0.0833	0.2038	2.2260	0.6642
**2 HCl 0.05 mM**	0.0794	0.0999	0.0661	0.2219	0.0889	0.0767	0.0738	0.0885	0.1216	0.0935	0.0905	0.0749	0.0691	1.1816	0.3797
**3 HCl 0.05 mM**	0.0452	0.0716	0.0477	0.0882	0.0917	0.0472	0.0529	0.0696	0.0864	0.0397	0.0130	0.0601	0.0616	0.3230	0.1460
**4 HCl 0.05 mM**	0.0787	0.0490	0.0698	0.0763	0.0693	0.0586	0.0769	0.1082	0.0946	0.0370	0.0103	0.0654	0.0767	0.0983	0.1314
**5 HCl 0.5 mM**	1.1244	0.6759	0.9259	1.1511	0.9625	1.3295	1.0540	1.1386	1.0551	1.3751	0.0000	0.2761	1.0652	0.1386	1.4420
**6 HCl 0.5 mM**	0.0133	0.0589	0.0224	0.0608	0.0145	0.0042	0.0208	0.0182	0.0822	0.0357	0.0208	0.0643	0.0158	0.0555	0.4817

## Abbreviations

DIPEA	N,N-Diisopropylethylamine
DCM	Dichloromethane
ESI-TOF	Electrospray ionization time-of-flight
Fmoc	Fluorenylmethyloxycarbonyl protecting group
HOBT	Hydroxybenzotriazole
MALDI-TOF	matrix assisted laser desorption and ionization time-of-flight
MES	2-(N-morpholino)ethanesulfonic acid
OtBu	tert-butyl ester group
Pbf	2,2,4,6,7-pentamethyldihydrobenzofuran-5-sulfonyl group
Pi	Phosphate
PPi	Pyrophosphate
TBTU	2-(1H-Benzotriazole-1-yl)-1,1,3,3-tetramethylaminium tetrafluoroborate
TCA	trichloroacetic acid
TFA	trifluoroacetic acid
Tris	tris(hydroxymethyl)aminomethane
Trt	trityl group
standard one-letter and three-letter codes for amino acids are used throughout the paper	
